# Rolling out PRIDE in All Who Served: Barriers and Facilitators for Sites Implementing an LGBTQ+ Health Education Group for Military Veterans

**DOI:** 10.1007/s11606-023-08204-5

**Published:** 2023-06-20

**Authors:** Sarah M. Wilson, Abigail C. Mulcahy, Tiffany M. Lange, Madeleine R. Eldridge, Hollis J. Weidenbacher, George L. Jackson, Jennifer M. Gierisch, Matthew J. Crowley, Patrick S. Calhoun, Michelle M. Hilgeman

**Affiliations:** 1 Mental and Behavioral Health Service, Durham VA Healthcare System, Durham, NC USA; 2 Center of Innovation to Accelerate Discovery and Practice Transformation (Health Services Research and Development), Durham VA Healthcare System, Durham, NC USA; 3 Department of Psychiatry & Behavioral Sciences, Duke University School of Medicine, Durham, NC USA; 4 Department of Population Health Sciences, Duke University School of Medicine, Durham, NC USA; 5 Center to Improve Veteran Involvement in Care (Health Service Research and Development), Portland VA Healthcare System, Portland, OR USA; 6 School of Public Health, Oregon Health and Science University/Portland State University School of Public Health, Portland, OR USA; 7 Community Behavioral Health, Philadelphia, PA USA; 8 Peter O’Donnell Jr. School of Public Health, University of Texas Southwestern Medical Center, Dallas, TX USA; 9 Department of Medicine, Duke University School of Medicine, Durham, NC USA; 10 Tuscaloosa VA Medical Center, Tuscaloosa, AL USA; 11 Department of Psychology, University of Alabama, Tuscaloosa, AL USA

## Abstract

**Background/Objective:**

The Veterans Health Administration (VHA) PRIDE in All Who Served health education group (PRIDE) was developed to improve health equity and access to care for military veterans who are lesbian, gay, bisexual, transgender, queer, and/or other sexual/gender-diverse identities (LGBTQ+). This 10-week program rapidly spread to over 30 VHA facilities in 4 years. Veterans receiving PRIDE experience improved LGBTQ+ identity-related resilience and reductions in suicide attempt likelihood. Despite PRIDE’s rapid spread across facilities, information is lacking on implementation determinants. The current study’s goal was to clarify determinants of PRIDE group implementation and sustainment.

**Methods:**

A purposive sample of VHA staff (*N* = 19) with experience delivering or implementing PRIDE completed teleconference interviews January–April 2021. The interview guide was informed by the Consolidated Framework for Implementation Research. Rapid qualitative matrix analysis was completed with methods to ensure rigor (e.g., triangulation and investigator reflexivity).

**Results:**

Key barriers and facilitators of PRIDE implementation were heavily related to facility inner setting (what is happening inside the facility), including implementation readiness (e.g., leadership support for LGBTQ+-affirming programming, access to LGBTQ+-affirming care training) and facility culture (e.g., systemic anti-LGBTQ+ stigma). Several implementation process facilitators enhanced engagement at sites, such as a centrally facilitated PRIDE learning collaborative and a formal process of contracting/training for new PRIDE sites.

**Discussion/Conclusion:**

Although aspects of the outer setting and larger societal influences were mentioned, the majority of factors impacting implementation success were at the VHA facility level and therefore may be more readily addressable through tailored implementation support. The importance of LGBTQ+ equity at the facility level indicates that implementation facilitation should ideally address institutional equity in addition to implementation logistics. Combining effective interventions with attention to local implementation needs will be required before LGBTQ+ veterans in all areas will benefit from PRIDE and other health equity-focused interventions.

**Supplementary Information:**

The online version contains supplementary material available at 10.1007/s11606-023-08204-5.

## INTRODUCTION

United States (US) military veterans who are lesbian, gay, bisexual, transgender, queer, and/or other sexual/gender-diverse identities (including but not limited to questioning, pansexual, asexual, agender, gender diverse, nonbinary, gender-neutral, and other identities; LGBTQ+) are a historically disenfranchised and currently underserved group within the Veterans Health Administration (VHA).^1,2^ Despite considerable progress in detecting and understanding LGBTQ+ health inequities.^3–5^ access to equitable and effective care is not yet consistent across VHA facilities.^1^ Common barriers have included discrimination, cisheteronormativity, limited electronic health record infrastructure for documenting sexual orientation and gender identity, and lingering effects of unjust policies that excluded LGBTQ+ individuals from military service.^6–11^

In 2016, VHA policy established designated LGBTQ+ Veteran Care Coordinators (VCCs) at each facility, which has paved the way for innovation by VHA staff committed to increasing access to affirming care.^12,13^ LGBTQ+-affirming care entails healthcare for LGBTQ+ patients that is culturally responsive and works to alleviate health inequities in this population through strategies such as creating a welcoming environment, addressing provider bias, providing tailored health services, and acknowledging underlying systemic inequality.^13^ Despite gains in the availability of LGBTQ+-affirming services within VHA,^14–16^ there is still a complete lack of affirming care interventions at many VHA facilities.^17^

In order to address this gap in LGBTQ+-affirming care offerings at VHA facilities, the PRIDE in All Who Served group intervention (PRIDE) was developed and spread by the PRIDE National Diffusion Team.^18–20^ This was made possible by the VHA Innovators Network, which links VHA facilities with the goal of helping frontline VHA staff develop innovative ideas to enhance VHA services.^21,22^ PRIDE has been designated as a National Diffusion Practice by the VHA Diffusion of Innovation program, which supports the spread of innovative VHA practices.^23^

The PRIDE intervention is a 10-week, structured health education group that focuses on social connection, health promotion, minority stress reduction, and engagement with available VHA resources.^19^ Significant reductions in self-reported suicide risk and symptoms of distress (e.g., depression) have been observed after attending the group as well as increases in protective factors (e.g., identity acceptance).^19,24^ Leading up to the current study, PRIDE rapidly spread from a single clinician at one facility in 2017 to being delivered at VHA facilities across the country in 2021. The PRIDE National Diffusion Team used external implementation facilitation (i.e., collaborative problem solving/support through a designated support person ^25^ as the overarching implementation strategy, with Fortney’s Access model (26) informing evaluation of the Veteran experience (e.g., perceived access) and health impact.^19^

As a result of this organic spread, PRIDE groups have reached more than 700 LGBTQ+ veterans.^20^ Yet, with less than 25% of VHA facilities currently delivering the program and less than 1% of the estimated LGBTQ+ veterans reached, a deeper understanding of factors that impact implementation and sustainment is needed to scale the program beyond the early adopting sites.^27,28^

This article qualitatively examines the facilitators and barriers that impact implementation, sustainment, and ultimately veteran access to the PRIDE intervention, including factors associated with shifting to virtual delivery during the first year of the COVID-19 pandemic.

## METHOD

### Study Design and Guiding Framework

Retrospective and prospective exploratory descriptive qualitative methodology and rapid qualitative analysis were used to clarify determinants of implementation across 18 sites using ^1^ existing facilitator field notes from previous site visits; and ^2^ key informant interviews. Study methodology (interview guide and analytic strategy) and interpretation of findings were guided by two implementation frameworks: the Consolidated Framework for Implementation Research (CFIR) and the Health Equity Implementation Framework (HEF).^29,30^ CFIR is a well-accepted framework for implementation science research and is particularly useful in guiding formative implementation research. The HEF complements CFIR in this context given its specific focus on using implementation science to decrease health disparities rooted in systemic inequity. Similar to CFIR, the HEF focuses on multiple levels of implementation determinants, including societal influence (i.e., wider systemic inequality), outer/inner context, and the clinical encounter.

### Participants and Recruitment

Prospective participants (*N* = 29) were PRIDE site implementation leads and members of the PRIDE learning collaborative from 29 sites. These prospective participants were sent a personalized secure VA email inviting participation in key informant interviews. Prospective participants were informed of the purpose of the study, and that participation was voluntary, one-time, and confidential. The sample size was a priori determined to be *N* = 20, since this number of interviews (given the relatively narrow research question) was likely to lead to data saturation.^31,32^ Participant inclusion criteria were being a VA staff member in one of the following roles: facility PRIDE implementation lead, PRIDE group leader, and/or local clinic/organizational leader familiar with PRIDE. For participants who agreed to study participation, informed consent was completed via encrypted email, with consent forms signed via pdf digital signature. Participants were offered an opportunity to ask any questions to a project staff member prior to digitally signing the consent form. Among prospective participants who expressed interest in participating, none refused participation. After participation, each consented participant was asked to identify up to three additional staff who could be approached for participation.

### Procedures

#### Site Field Notes

 As part of a VHA Innovators Network Spread quality improvement grant prior to the current study (mPIs TL and MH), TL completed external facilitation site visits and recorded semi-structured field notes for each site. The site visits (which some sites did not receive in-person due to the COVID-19 pandemic) included a training in the PRIDE manual for group leads, a training in LGBTQ+-affirming care open to all staff at the facility, and a facilitated meeting with site leadership to promote the rationale and importance of LGBTQ+-affirming care and the PRIDE group. Field notes detailed items such as visibility of LGBTQ+-affirming care symbols, hospital leadership engagement in site visit, and other clinic- and provider-level factors. Although these field notes were not originally created for research, for the current study we obtained IRB approval to use field notes to triangulate analysis with participant interviews (see the “Analysis” section below).

#### **Participant Interviews**

Consented participants completed a semi-structured qualitative interview, which was audio recorded and transcribed by professional transcriptionists external to the team. Interviews lasted on average 48 min. Given the small study sample size and small pool of prospective participants, participants were not asked to report demographic information in order to preserve their anonymity and to alleviate concerns about reprisal for candid responses.^33^

The interview guide (see Appendix 1) was piloted internally in the research team prior to use with participants. The interview guide focused on describing facilitators and barriers of implementing the PRIDE intervention, and was broken down loosely into sections according to four CFIR/HEF domains: Intervention Characteristics, Implementation Process, Inner Setting (what is happening inside the specific VHA facility), and Outer Setting/Societal Influence (what is happening outside the specific VHA facility in places such as the local community or broader VHA).^29^ All study procedures were approved by the Durham VA Health Care System Institutional Review Board.

### Analysis

Due to the quick timeline of this 1-year pilot study and the goal of informing ongoing implementation work, a rapid qualitative analysis approach was used to analyze the data.^34–36^ Microsoft Excel and Word were used to complete this process. Three team members (SW, ME, MH) developed and used a template to summarize the first three transcripts and develop consistency across the analysis team. A summary of each transcript was then created using the template, before further condensing the data using matrix analyses by case and CFIR/HEF domain. Finally, tables were created of each barrier and facilitator with consensus definitions and mapping back to the CFIR/HEF domains and subconstructs.

For site visit field notes, a matrix was created of site by existing field note sections (trainings provided, implementation facilitators, implementation barriers, LGBTQ+ visibility, additional tasks, and miscellaneous comments). The matrix of site visit field notes was triangulated with the matrix of themes from the key informant interviews to generate a comprehensive understanding of implementation determinants. Reflexivity memos and an audit trail were among the methods used to ensure rigor during design and analysis stages.

### Investigator Reflexivity

The research team reflected gender diversity (cisgender, demigender, neutrois, and nonbinary) and sexual orientation diversity (bisexual, pansexual, queer, and straight). The team all had advanced degrees and was majority White, but reflected racial diversity in its leadership (Black, mixed race, and White leadership). At the time of the study, the team all had roles in either health services research or healthcare innovation. TL, MH, and SW all had prolonged engagement with prospective participants that began prior to initiation of the study. TL was the creator of the PRIDE intervention. TL and MH led spread and implementation of PRIDE. SW was a former site lead for the PRIDE intervention. The team’s (TL, MH, SW) lived experience of PRIDE implementation was allowed to enhance the research process (e.g., interview guide, qualitative analysis, interpretation). The qualitative analytic team (ME, MH, and SW) all consider themselves to be LGBTQ+ advocates. The qualitative interviewer (ME) had no previous contact with prospective participants and was a master’s trained qualitative analyst with experience in data collection and content analysis.

## RESULTS

**Figure 1:**
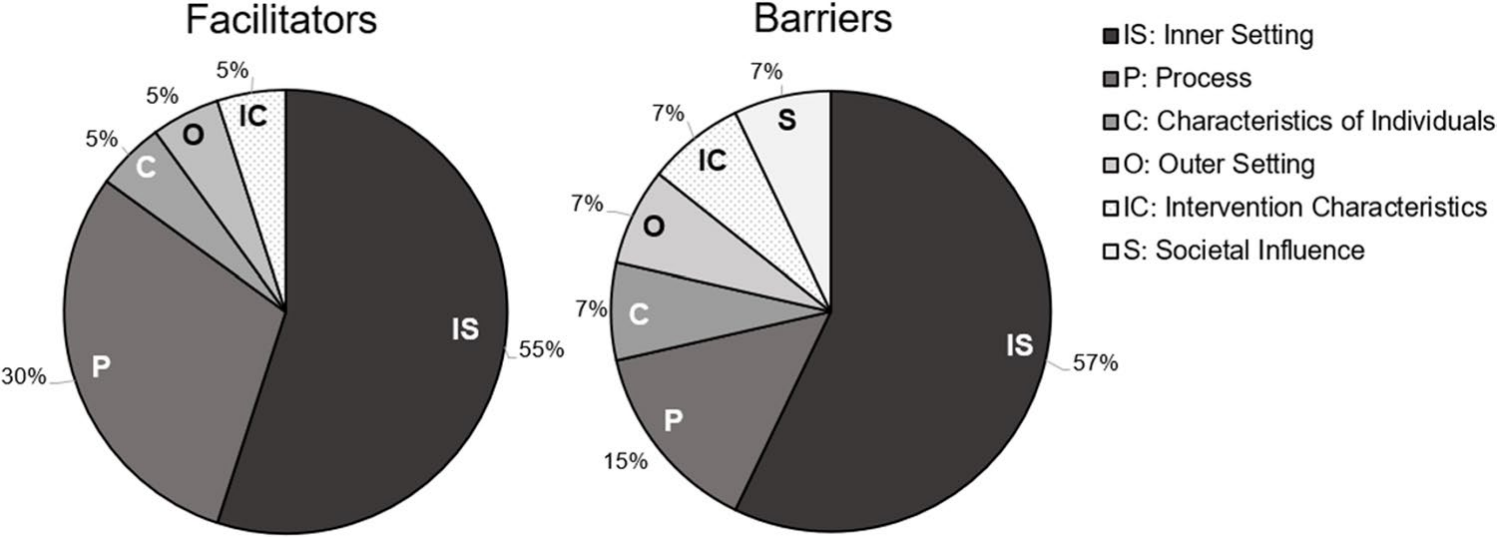
Synthesis of barriers and facilitators into domains.

A total of 20 staff participants consented to the study. One consented participant was lost to contact prior to completing an interview, leaving 19 staff participants from 18 sites who completed structured interviews. See Table 1 for site characteristics. Sites displayed a variety of PRIDE implementation stages (see Table 1). Field notes from 10 site visits to VA facilities were also analyzed. Figure 1 shows a graphic depiction of the specific CFIR/HEIF domains that corresponded to barrier- and facilitator-related themes. The majority of themes mapped onto Inner Setting and Process CFIR domains.

**Table 1:** Site Characteristics, *N* = 18

	*N* (%)
US geographical region	
South	9 (50.0%)
West	5 (27.8%)
Midwest	4 (22.2%)
Northeast	0 (0.0%)
PRIDE implementation stage	
Pre-implementation	3 (16.7%)
Launched one cohort	8 (44.4%)
Completed one or more cohorts	8 (38.9%)
2020 Healthcare Equality Index rating*	
Leader status	4 (22.2%)
Top performer status	10 (55.6%)
Submitted, other score	0 (0%)
Not submitted	4 (22.2%)
Facility-level percent rural patients	
0 to 24% rural	5 (27.8%)
25 to 49% rural	12 (55.6%)
50% or more rural	3 (16.7%)

*The Healthcare Equality Index is a biannual survey of health care systems nationwide that summarizes LGBTQ-inclusive patient, visitation, and employment policies. Facilities are either classified as Not Submitted or fall under one of three tiers (Leader, Top Performer, or Submitted)

### Barriers to PRIDE Implementation

Rapid qualitative analysis yielded 7 barrier-related themes (see Appendix 2 for definitions of barrier themes), which mapped onto 14 CFIR/HEIF domains (some themes mapped onto multiple domains). Themes below include quotations and anonymized ID codes.

#### **Themes Relating to Setting Up and Completing the Clinical Encounter**

Themes relating to clinical care included Navigating Virtual Group Delivery and Issues Running Group Sessions. Overall, these themes fell under four CFIR domains: Outer Setting (patient needs for participation), the Inner Setting (available resources for implementation), the Process (executing the group itself), and Intervention Characteristics (the complexity of the intervention due to its group telehealth format). Due to the COVID-19 pandemic, national VA policy changes forced a switch from in-person to virtual care; for the PRIDE group leaders, this rapid change to video telehealth groups highlighted gaps in access and willingness of veterans to engage in virtual groups. In the words of one participant (8282), “I think a lot of our Veterans don’t like the virtual format. And if it was in-person, I think we would have more attendance.” PRIDE group leaders also noted challenges in running the group due to low engagement, high attrition, and/or interpersonal conflicts between veterans attending group. One participant (8574) noted, “It’s difficult to have a closed group [i.e., not open to enrollment after the start of the group] and expect people to show up regularly. [… I was] feeling pressured to not have somebody wait for the next round of the group but offer them that as a resource.”

#### **Themes Relating to Organizational Support and Work Relationships**

Three themes related to this topic: Lack of Support from Leadership, Needing to Work with or Rely on Others as a Problem, and Issues with Facilitator Time and Role Definition. Themes relating to organizational support and work relationships fell under two CFIR domains: Inner Setting (leadership engagement, networks and communication, resources available for implementation, and implementation climate) and Characteristics of Individuals (knowledge/beliefs about the intervention). Overall, these themes highlighted that group implementation and sustainment were negatively impacted by lack of support from clinic leads, facility leadership, peer clinicians, and clerical staff. Site implementation leads often felt they had to take an unaided and solitary approach to be successful. One participant (8966) voiced this as a challenge with “getting people to move at your speed and getting people to kind of feel like something is important.”

#### **Infrastructure and Logistics**

This theme related to the CFIR domains Inner Setting (resources available for implementation) and Process (executing the group itself). This theme also links back to the two themes relating to clinical care and organizational support. Some site leads lacked clinic leadership support, protected time to work on setting up the group, and clerical support; this lack of support negatively impacted their ability to navigate the logistics of setting up the PRIDE group. Difficulty with finding referrals also fell under this theme, which often linked back to a lack of support from colleagues to expend effort to help identify veterans who would benefit from the group (e.g., “Advertising was not going well. I had one person who wanted to participate, so I completed it as an individual manualized therapy.” Participant 8712). Participants felt that there were LGBTQ+ veterans receiving care at their facility—they just noted infrastructure barriers to identifying these veterans and referring them to the PRIDE group.

#### **Discrimination and Systemic Oppression**

This theme related to the HEIF domain Societal Influence (sociopolitical forces) and the CFIR domain Inner Setting (workplace culture). Effects of systemic discrimination appeared at the societal level (LGBTQ+ stigma), structural level (few providers designated as LGBTQ+ affirming), interpersonal level (anti-LGBTQ+ materials left in hospital waiting rooms), and individual level (veteran self-stigma). For example, one participant (8886) noted, “Brochures would disappear. I’d put a bunch of pamphlets out and three days later they were all gone. […] We’ve had some struggle with providers not following [LGBTQ+-affirming] policies and that kind of thing. […] I think that there’s a fairly [large] proportion of providers that [LGBTQ+ health] is not really an issue that they care much about.”

Of note, all participants were interviewed soon after Executive Order 13950 (“Combating Race and Sex Stereotyping,” sometimes referred to as the ban on diversity training) was released by the White House on September 22, 2020. Although this executive order did not apply to any of the PRIDE group content or staff trainings, some study participants perceived a deterring effect of this executive order on recruiting for and running the PRIDE group. For example, one participant (8975), stated: “The Chief of Mental Health and the Chief of Staff went over our PowerPoints from [the PRIDE implementation facilitator]. […] We took out all the language that was objectionable according to the executive order.” (Please note that none of the language within the PRIDE implementation materials technically should have needed to be removed, as it complied with Executive Order 13950.)

This theme also encompassed feelings of exhaustion among PRIDE site leads surrounding effort and time taken for LGBTQ+ advocacy in environments without sufficient supports. For example, a participant (8168) stated:

The culture here is kind of awful.[…] It just became more and more clear that it’s just a civilized surface and a ton of bigotry and discrimination underneath. And I used to think that having a group and encouraging people to understand why it’s important to be honest with your providers and all that was, you know, a step towards culture change. But now I just want them to be safe. And it’s hard to be safe when your record is full of progress notes that say ‘LGBT Group.’ It’s just not safe here.

### Facilitators of PRIDE Implementation

Analysis yielded 6 facilitator-related themes (see Appendix 3 for definitions of facilitator themes), which mapped onto 20 CFIR/HEIF domains (some themes mapped onto multiple domains).

#### **Themes Relating to the PRIDE National Diffusion Team**

This topic related to three themes: Strong Base of Materials; Training, Knowledge Transfer, and Clinician Consultation; and Infrastructure for Shared Learning Across Sites. Themes in this topic area mapped onto three CFIR domains: Intervention Characteristics (design quality and packaging), Inner Setting (resources available for implementation, access to knowledge, and information, learning climate), and Process (external change agents, learning collaborative, formally appointed implementation leaders). Overall, these themes highlighted the successes of the implementation strategies used in terms of helping the site leads feel supported and prepared to start and maintain the group. The design and content of the PRIDE group manual and handouts were appealing to site leads and group leaders. Moreover, the national PRIDE external implementation facilitator (Dr. Lange) was perceived as a source of knowledge and empowerment. For example, one participant (8460) noted,

[The PRIDE implementation facilitator] kind of served like a backbone to the whole project. She was very good about ensuring that if we came across any lack of support from leadership or things at our own facility, that she would be willing to kind of step in. And we didn’t need her to do that, but I think that also just kind of empowered us to feel more confident in the choices we were making in continuing the group.

#### **Themes Relating to LGBTQ+ Collaboration and Training**

This topic area consisted of the following two themes: Intra-Facility LGBTQ+ Visibility, Collaboration, and Support; and Access to LGBTQ+ Training, Expertise, and Non-VA Community Organizations. These themes related to two CFIR domains: Inner Setting (culture, implementation climate, networks, and readiness for implementation) and Outer Setting (cosmopolitanism). Themes in this topic area tied into the need for facilitators to approach implementation of the PRIDE group with a foundation of knowledge and collegial/institutional support. This topic area also tied back to a counteracting force against the barrier theme Discrimination and Systemic Oppression. High levels of LGBTQ+ institutional visibility and LGBTQ+ expertise/training could somewhat counteract systemic LGBTQ+ oppression. One participant (8911) noted, “The [Health System] Director that we have now is very supportive of anything that I do. My department heads – they support what I do. So I don’t have any issues with anybody supporting my running the group, and there’s always people who want to be in and want to participate in the group.”

#### **Maintaining Local Gains by Working Together**

This theme reflected the importance of having strong relationships with colleagues, clinic leads, and facility leaders. This theme related to Inner Setting (networks and communication, readiness for implementation) and Process (executing, reflecting, and evaluating). This theme was in stark contrast to the barrier-related theme Needing to Work with or Rely on Others as a Problem. Within this theme, participants noted being able to have a team to support implementation of PRIDE. These participants found that relying on others was a support rather than a burden. One participant (8574) stated, “All of my colleagues are really on-board with posting visual safety items in their offices, which made it more comfortable, I think, for Veterans to kind of out themselves in the therapy room. And so having colleagues give me referrals and having my facility give me the space I need to create this group – that was really helpful.”

#### **Facilitators Among Sites Completing One or More Cohort**

Six of the participants from sites that completed one or more cohort of the PRIDE intervention noted themes relating to the positive effects of external facilitation. All but one of the participants from sustaining sites also noted support from either colleagues or leadership being important for implementation and/or sustainment.

## DISCUSSION

This study used rapid qualitative analysis to clarify implementation determinants of an LGBTQ-affirming health education group intervention for veterans. Findings indicated the importance of the inner setting CFIR domain in both barriers and facilitators of PRIDE implementation. These facility-level factors are important because they reflect the structure, culture, and communication present at VA facilities. Additionally, LGBTQ+ visibility, support, discrimination, and systemic oppression arose across multiple themes. These issues point to the ongoing unique needs and experiences of LGBTQ+ veterans accessing healthcare.

Although barriers to accessing care may be framed as either “actual” or “perceived,”^26^ this study’s findings suggest that may be an oversimplification. The use of the actual/perceived access dichotomy may inadvertently invalidate the legitimate anxieties about discrimination that both LGBTQ+ veterans and LGBTQ+-affirming staff may have. Obstacles to accessing LGBTQ+-affirming care exist at the individual, clinician, and systemic level, and in this study, we observed a blending of actual/objective and perceived/subjective barriers to accessing care. Regarding group referrals, for example, several participants endorsed difficulty with obtaining referrals, especially when the facility environment was unwelcoming or lacked support for logistics in initiating the group. Additionally, participants often had a passionate desire to promote health equity, but campaigning for reform is a known contributor to advocacy burnout among professionals.^37,38^ Site leads reported lack of protected time, resources, and internal support, which not only led to implementation/sustainment barriers but may have contributed to exhaustion around advocacy efforts. Given varying levels of internal facility support for implementation, the availability of external facilitation from the PRIDE National Diffusion Team appeared to help overcome these actual/perceived barriers to PRIDE implementation.

This study also demonstrated the usefulness of the HEIF when combined with CFIR. The equity lens helped highlight how implementation of LGBTQ+-affirming interventions can be affected by anti-LGBTQ+ stigma, which can in turn burden LGBTQ+ veterans seeking healthcare. Although VHA has national policies that affirm LGBTQ+ veterans, there is variability in how these policies are implemented across facilities.^13^ As demonstrated in this study, it is imperative that LGBTQ+-affirming policies and healthcare innovations have adequate facility-level support to promote consistent implementation.

This study has three limitations. First, consistent with the qualitative design, generalizability was not a goal of this study. Instead, we focused on answering “how” questions in rich detail. Second, not all regions of the US were represented in this study. However, this mirrors the spread of the PRIDE intervention, which focused on spread to the South. Third, for the most part only one participant was interviewed per site, which may not be fully representative of the experience of implementing and sustaining the program. This study also has two key strengths: ^1^ interviews were triangulated with field notes to ensure reliability; and ^2^ to minimize bias, interviews were conducted by an interviewer who was not affiliated with PRIDE implementation.

## CONCLUSIONS

The current study clarified determinants of implementation and sustainment of an LGBTQ+-affirming educational group at 18 facilities within the VHA. Since the conclusion of the study, the PRIDE intervention has now been delivered at 42 VHA facilities across the country with an additional 17 VHA facilities currently preparing to implement the group for the first time.^39^ Further spread of the PRIDE intervention will ensure equitable veteran access to this innovative program. Based on the study findings, there are four key recommendations for sites seeking to improve implementation or sustainment of PRIDE: (1) solicit support from leadership early in the implementation process, (2) build collaborative teams of LGBTQ+-affirming staff at and outside of the facility, (3) use and refer back to PRIDE implementation materials, and (4) discuss and address institutional intersectional equity. Future work may link determinants of implementation to potential targeted strategies for PRIDE site implementation or investigate effects of unequal power differentials in implementation of equity-focused interventions.

## Supplementary Information

Below is the link to the electronic supplementary material.

Supplementary file1 (DOCX 25 KB)

## Data Availability

The dataset generated during the current study is not publicly available due to the need to protect the confidentiality of the staff participants in the study. However, a deidentified dataset (with potential contextual identifiers removed from interview transcripts) is available from the corresponding author on reasonable request and completion of a data use agreement.
