# Supplementary material for: Rolling out PRIDE in All Who Served: Barriers and Facilitators for Sites Implementing an LGBTQ+ Health Education Group for Military Veterans
Source: J Gen Intern Med. 2023 Jun 20;38(Suppl 3):849–56. doi: 10.1007/s11606-023-08204-5 (PMC10356690; doi:10.1007/s11606-023-08204-5)
Supplement: Supplementary file 1 — Supplementary file1 (DOCX 25 KB) [file 11606_2023_8204_MOESM1_ESM.docx]

**Appendix 1** Interview Guide

Note: This is an interview guide for qualitative interviews of participants as laid out in the study protocol. The interviewer will use this guide to organize the interview. Because it is a guide, exact questions may vary during the course of the interview or order may vary somewhat depending on answers given by participants. However, the guide provides a general structure and order for questions and ensures that all domains are covered in the interview as appropriate, based on the answers of the participant being interviewed. The interviewer will adhere to the guide, but will also follow the conversation, which may stray from the guide as he/she feels is appropriate

| **Orientation to the Interview**  Thank you for taking the time to speak with me today. I’m (Name), a(n) (role) with the Durham VA Health Care System, and I am going to be asking you questions about PRIDE in All Who Served. You participated in the implementation of PRIDE in All Who Served at your site, (SITE). We want to learn about barriers and facilitators you experienced, and about your overall experience with program implementation. This interview will last approximately 45 minutes to an hour. I’m looking forward to hearing your thoughts.  What questions do you have before we begin?  The consent form stated that your interview would be audio recorded. I wanted to remind you of that before we get started. Are you still alright with audio recording this interview?  If yes: Thank you.  Please let me know if at any point you want me to turn off the recorder.  If no: Okay, let’s continue without the recorder.  *Note to Interviewer: This interview is guided by the Consolidated Framework for Implementation Research (CFIR; see* [*https://cfirguide.org/*](https://cfirguide.org/) *for more information).* |
| --- |
| **Intervention Characteristics Section**  *This section focuses on the participant’s perspective on key elements/features of the PRIDE intervention that may influence adoption and sustainment.*  First, we’ll discuss the different parts of the PRIDE in All Who Served Group. I’d like to briefly review the manual together [*share screen with one-page session overview*]: Pride in All Who Served: A Health Education Support Group for LGBT Veterans (called the Pride Group, for short; Lange-Altman, 2018) is a manualized program of veteran-focused content, psycho-educational handouts and resources, self-reflection exercises/tools, and guided discussion questions that encourages group-level processing. Weekly topics include: (1) continuums of identity, LGBT terminology, and definitions, (2) coming out, emergence, and disclosure, (3) identity models, (4) military culture, (5) VA culture, (6) affirmative care and whole health, (7) sexual health, (8) healthy intimate relationships, (9) LGBT families, and (10) community resources and conclusions.  1. From your perspective, what are the most important key or core components of the PRIDE group manual? (What makes [component] important?)  2. [After briefly reviewing manual] What adaptations or changes were made to the PRIDE group from the manual at your site?  Probe: What were the reasons that these adaptations were made?  3. From your perspective, what components of the PRIDE group manual are optional? (What makes [component] optional?) |
| **Implementation Process Section – Executing and Reflecting**  *This section focuses on the different stages and activities involved in implementation and sustainment of the intervention.*  Next, we want to know about your experience in attempting to start and run the Pride group.  4. Walk me through the steps that were taken at your VA facility to get the Pride group started.  Probe: What tools, trainings, and resources did you receive that were helpful in starting the PRIDE group?  Probe: What challenges did you face in getting the group started?  Probe about site visits: Did you have a facilitation site visit from Dr. Lange? (If so, how did that facilitation site visit affect your experience of starting and sustaining the group?)  5. How successful would you say that your facility was in maintaining or sustaining the PRIDE group?  Probe: What were the reasons that you were able to/not able to maintain the PRIDE group?  6. How has the COVID-19 pandemic impacted your ability to start or maintain the PRIDE group?  Probe: How might these COVID-19-related challenges be overcome or managed? |
| **Implementation Process Section – Evaluating**  *This section focuses on potential effectiveness and implementation outcomes associated with the intervention.*  We also want to know about Veteran experiences:  7. What experience did Veterans have joining and participating in the Pride group at your facility?  Probe: What, if anything got in the way of Veteran participation in the group?  Probe: How was the group received by Veterans?  Probe: What impact, if any, did the PRIDE group have on Veterans?  Anchoring statement: We’re about halfway through my questions:  Let’s continue reflecting on your experience in setting up and maintaining the PRIDE group:  8. What changes did you notice within your clinic or health care system after implementing the PRIDE group?  Probe: In what way (if at all) was the PRIDE group related to this change?  Probe: In what way (if at all) was the facilitation site visit related to this change?  9. **(Skippable)** What advice would you give to a VA staff member who is trying to start and maintain a regularly recurring PRIDE in All Who Served group at their VA facility?  Probe: What makes a good PRIDE facilitator?  Probe: What sort of experience do they need? |
| **Inner & Outer Setting Section**  *This section focuses on describing the local VA facility and local community cultural context related to LGBT-affirming care. We are interested in what ways local context may affect implementation of the intervention.*  Next, let’s talk about how LGBT affirming your VA facility has been in the past and is now.  10. Before you started the PRIDE group, what level of LGBT+ affirmation/visibility was present within your VA facility? (Can you describe what that looked like?)  Probe: In what ways did your local VA facility culture affect your experience of starting and sustaining the group?  11. Currently, what is the level of LGBT affirmation/visibility present within your VA facility? (Can you describe what that looks like?)  Let’s also talk about the local community outside of your VA facility, and the local culture.  12. Before you started the PRIDE group, what level of LGBT visibility was present in the wider community beyond your VA facility? (Can you describe what that looked like?)  **(Skippable)** Probe: In what ways did your local community and culture influence your experience of starting and sustaining the group?  Probe: Have you noticed any changes since implementing PRIDE?  Probe: In what ways did your local community and culture influence how the group was received by Veterans? |
| **Closing Questions**  (Anchoring statement): We’re very nearly finished:  14. Could you identify and briefly describe the role of a few people (staff) you think we should interview about PRIDE?  15. Is there anything you’d like to mention that I didn’t ask you about? |

**Appendix 2** Barriers to Implementation of the PRIDE Group

| **Theme** | **Theme description** |
| --- | --- |
| Navigating Virtual Group Delivery | Challenges linked to the following: starting a virtual group or switching from in-person to virtual group delivery, veteran access to technology, and veteran willingness to engage virtually. |
| Issues Running Group Sessions | Difficulty in the following: identifying and recruiting enough veterans for a group, finding a meeting time that works for all interested veterans, maintaining engagement and attendance in the group, and managing emotional content and conflict within sessions. |
| Lack of Support from Leadership | Facilitator perceptions of the following: lack of assigned administrative support, LGBTQ+ health not being a priority with service line chiefs and/or facility leadership, and insufficient protected time for PRIDE group leaders. |
| Needing to Work with or Rely on Others as a Problem | Challenges and frustrations linked to needing help or resources from other VA staff members, but not feeling able to trust in others’ willingness or availability to help. |
| Issues with Facilitator Time and Role Definition | Other priorities competing with time to the following: create, promote, facilitate, and complete administrative tasks for the group. These tasks were perceived as essential to group success, yet there was often insufficient time for them. |
| Infrastructure and Logistics | Facilitators faced challenges around setup (e.g., creating clinics and note titles, getting veteran referrals, mailing handouts). It could also be difficult to find a clinical service home for the group since it was a health education group. |
| Discrimination and Systemic Oppression | Facilitators observed the effects of systemic oppression, ranging from hesitancy to discuss sexual orientation and gender identity in clinical care settings, to recruitment materials being pulled down and thrown away, to administrative staff having concerns about advertising an LGBTQ+ group. Transphobia was highlighted in this theme. |

**Appendix 3** Facilitators to Implementation of PRIDE Group

| **Theme** | **Theme description** |
| --- | --- |
| Strong Base of Materials | Facilitators noted the helpfulness of supporting materials for implementing PRIDE, like the group manual (including group setup instructions and session content), example recruitment fliers, workbooks, and materials that created a welcoming atmosphere (e.g., rainbow stickers, lanyards). |
| Training, Knowledge Transfer, and Clinician Consultation | Site facilitators found the national PRIDE external implementation facilitator to be a powerful source of support, through the site visits, trainings, provision of guidance and advice, and as someone who would advocate with leadership if needed |
| Intra-Facility LGBTQ+ Visibility, Collaboration, and Support | Facilitators described the power of leadership support, the support of clinic team members who worked together with internal facilitators on the PRIDE group, the support of staff who provided clerical help and expertise, and having visible LGBTQ+ staff. |
| Maintaining Local Gains by Working Together | Things that helped maintain the PRIDE Group included having a team of others to work with, leadership support, veteran enthusiasm for the group, and technology for overcoming distance as a barrier. |
| Access to LGBTQ+ Training, Expertise, and Non-VA Community Organizations | Facilitators described both existing access to LGBTQ+ education and seeking out additional resources for education. These resources included Fenway trainings, textbooks, articles, listservs, and WPATH guidelines, LGBTQ+ subject matter experts, and local/national community agencies (e.g., LGBTQ+ centers). |
| Infrastructure for Shared Learning Across Sites | The presence of a learning collaborative with options for consultation from the national PRIDE external facilitator helped boost motivation to facilitate the group, answer questions, and problem-solve. |
